# Shopping Data for Population Health Surveillance: Opportunities, Challenges, and Future Directions

**DOI:** 10.2196/75720

**Published:** 2025-08-06

**Authors:** Alisha Suhag, Romana Burgess, Anya Skatova

**Affiliations:** 1 Digital Footprints Lab & Medical Research Council Integrative Epidemiology Unit, MRC Integrative Epidemiology Unit, University of Bristol, Oakfield House United Kingdom, Bristol, BS8 2BN, United Kingdom, 44 (0)117 331 0098

**Keywords:** epidemiology, shopping data, health behaviors, diet, smoking, alcohol, surveillance, digital footprint data

## Abstract

The growing ubiquity of digital footprint data presents new opportunities for behavioral epidemiology and public health research. Among these, supermarket loyalty card data—passively collected records of consumer purchases—offer objective, high-frequency insights into health-related behaviors at both individual and population levels. This paper explores the potential of loyalty card data to strengthen public health surveillance across 4 key behavioral risk domains: diet, alcohol, tobacco, and over-the-counter medication use. Drawing on recent empirical studies, we outline how these data can complement traditional epidemiological data sources by improving exposure assessment, enabling real-time trend monitoring, and supporting intervention evaluation. We also discuss critical methodological challenges, including issues of representativeness, data integration, and privacy, as well as the need for robust validation strategies. By synthesizing the current evidence base and offering practical recommendations for researchers, this paper highlights how loyalty card data can be responsibly leveraged to advance behavioral risk monitoring and support the adaptation of epidemiological practice to contemporary digital data environments.

## Introduction

Noncommunicable diseases (NCDs) account for over two-thirds of global mortality [1]. Addressing 4 primary modifiable risk factors—tobacco use, unhealthy diet, physical inactivity, and alcohol consumption—could prevent up to 80% of major NCDs [2]. However, monitoring these evolving risk factors at scale remains a major challenge in epidemiology.

Traditional data sources—such as self-reported surveys, health registries, and administrative records—are crucial for population health monitoring but increasingly limited by high costs, slow survey cycles, and reporting biases (eg, recall and social desirability), reducing their reliability for tracking dynamic health behaviors. Participation in cohort studies and epidemiological surveys is also declining, raising concerns about selection bias and representativeness [3,4]. This is especially critical in research on lifestyle-related risk factors, where nonresponse often correlates with confounders such as socioeconomic status and health care access, contributing to spurious associations [5,6]. Lower participation rates among disadvantaged groups—who bear a disproportionate burden of NCDs—may further distort assessments of health inequalities [7,8]. In parallel, broader technological shifts and declining public engagement with surveys have compounded these challenges [9].

As traditional data sources struggle to capture the complexity and pace of population health dynamics, researchers are increasingly leveraging digital data—such as data from retail transactions, mobile apps, and other web-based activities. This shift reflects the growing momentum of digital epidemiology, which shares the foundational goals of traditional epidemiology—understanding and improving health at the population level—but sets itself apart by using data not originally generated for epidemiological purposes [10]. One such data resource is supermarket loyalty card data, offering objective, high-frequency insights into population health behaviors.

## Loyalty Card Data: A Tool for Epidemiological Research

Among the vast streams of data generated daily, supermarket loyalty card data—purchase records collected at checkout—provide granular, population-level insights into consumption behaviors relevant to public health [11]. Capturing details on purchased products, transaction frequencies, and store locations, loyalty card data can serve as a cost-effective research tool, as retailers already collect these data. Originally developed for retail marketing, these data are now being repurposed for public health research under strict General Data Protection Regulation (GDPR) guidelines, ensuring deidentified data access and confidentiality [12].

Loyalty card data have several advantages for epidemiological research. First, it provides an objective record of purchases**,** reducing recall errors and social desirability bias common in self-reported surveys. Second, with widespread adoption across multiple countries [13,14], loyalty cards grant access to large and diverse population samples. Third, as purchase histories—spanning food, health products, and other nondurable goods—are continuously recorded, often across decades, these data enable longitudinal tracking of short- and long-term trends, seasonal variations, and emerging health patterns with a level of granularity and temporal precision rarely achievable through conventional epidemiological data sources.

Demographic metadata from loyalty card programs allows researchers to link purchasing data with existing health datasets (eg, electronic health records, longitudinal surveys) [15,16]. This is particularly useful for studying lifestyle-related risk factors that are difficult to assess via self-reporting, such as moderate tobacco use or seasonal alcohol consumption. Geospatial data—such as store locations and customer postcodes—support spatial analyses of risky behaviors [17], helping identify regional disparities in food access, exposure to harmful products, and environmental influences.

Nonetheless, like any emerging data source, loyalty card data pose methodological challenges that must be addressed [13]. These include privacy concerns, data integration challenges, and methodological constraints. However, advancements in data science and data linkage protocols have enabled successful integration of loyalty card data in large-scale cohort studies such as Avon Longitudinal Study of Parents and Children, showing feasibility for broader implementation [16]. Such approaches are currently most applicable in high-income settings, where supermarkets dominate food supply chains and digital retail is well established [14,18]. As organized retail and mobile-linked loyalty schemes grow in low- and middle-income countries, analogous transaction data may become a scalable option for health surveillance.

## Aims

This paper explores the potential of supermarket loyalty card data as a viable data source for epidemiological surveillance, providing granular, high-frequency insights into population health behaviors. It highlights applications of loyalty card data across 4 key behavioral domains—diet, alcohol, tobacco, and over-the-counter (OTC) medications—and discusses key methodological considerations for leveraging these data in epidemiological research.

## Epidemiological Use Cases Across Key Behavioral Domains

**Figure 1.**
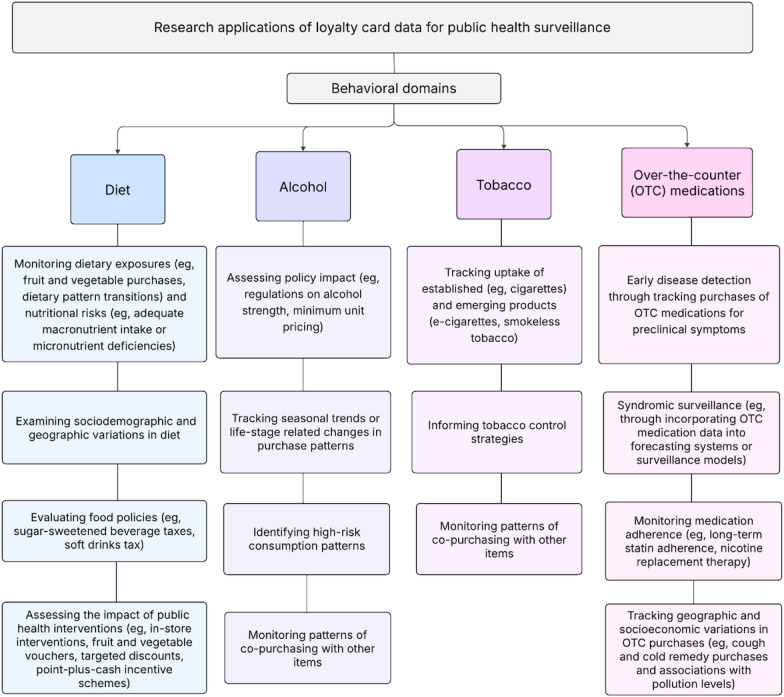
Research applications of loyalty card data across 4 behavioral risk domains.

Figure 1 provides an overview of some of the ways in which loyalty card data can be used to provide public health insights across diet, alcohol, tobacco, and OTC medicines.

### Diet

Diet is notoriously difficult to measure due to variations in both the types and quantities of food consumed. As a result, no single “gold-standard” method can address all diet-related questions [14]. Self-reported dietary assessments—such as food frequency questionnaires and 24-hour recall diaries—are limited by recall bias, underreporting, and logistical constraints (eg, limited diet-related questions in multitopic longitudinal studies) [19]. Given the complexity of diet and its outsized impact on health, supplementing self-reports is essential to address gaps in traditional dietary assessments.

Supermarket loyalty card data provide an objective, high-resolution record of food purchases [14] and can serve as a complementary resource for addressing key epidemiological questions: How do dietary habits and associated health outcomes vary by demographic and socioeconomic factors? How can emerging dietary risks or nutritional deficiencies be detected in real time? Are long-term dietary shifts associated with changes in the consumption of specific nutrients, or preferences for products with specific processing methods or ingredients? Table 1 illustrates the range of diet-related questions that supermarket loyalty card data can be used to address—from mapping sociodemographic differences in food purchasing, through validating purchase-based nutrient estimates, to evaluating fiscal or in-store interventions. These applications are discussed in greater detail in the sections that follow.

**Table 1.** Applications of loyalty card data in studying diet and nutritional intake.

Research themes and their applications	Research studies using loyalty card data
Dietary exposures	
Sociodemographic differences in dietary patterns	Identified dietary patterns using UK supermarket loyalty card data, linked to nutrient intake and socioeconomic characteristics [20].
Regional variation in produce purchases	Analyzed geographic variation in fruit and vegetable purchases using loyalty card data [21].
Purchase preferences of specific food groups	Identified clusters with different preferences in protein sources, including transitions away from red meat [22].
Longitudinal trends in purchasing patterns by sociodemographic characteristics	Modeled trends in produce purchasing over time, stratified by age and income groups [23].
Emerging micronutrient deficiencies	Detected population-level iodine deficiency risks linked to reduced dairy and increased plant-based milk purchases [24].
Comparison with self-reported data	
Validation of food purchases and survey data	Compared loyalty card–based protein purchases with self-reported intake among older adults to assess concordance [25].
Comparison of loyalty card data with self-reported consumption	Linked grocery purchases with food frequency questionnaire responses to examine variations across population subgroups [26].
Intervention effectiveness	
Assessing impact of voucher programs and financial incentives on consumption choices	1. Evaluated the impact of vouchers on fruit and vegetable purchases in low-income households [27]. 2. Evaluated a points-plus-cash program promoting fruit and vegetable purchases [28] 3. Investigated whether supermarket-based nudges and pricing changes affected ultraprocessed food purchases [29].
Impact of nutritional information interventions on food choice	1. Evaluated long-term effects of grocery store podcasts on omega-3 purchases [30]. 2. Assessed the impact of point-of-sale nutrition information on food selection [31].
Associations with health outcomes	
Effects of supermarket discounts on health outcomes	1. Studied effects of discounts on fruits, vegetables, and noncaloric beverages on weight loss and diet quality [32]. 2. Studied the real-world effects of supermarket nudging and pricing strategies and mobile physical activity coaching on diet quality, food-purchasing behavior, walking behavior, and cardiometabolic risk markers [33].
Food purchases and disease outcomes	Examined associations between loyalty card–based food purchases and health outcomes including hypertension, high cholesterol, and diabetes [34].
Policy impact assessment	
Sugar-sweetened beverage tax	Studied the impact of sugar-sweetened beverage tax effects on beverage purchases, with attention to subgroup-specific effects [35].
UK soft drinks sugar tax	Assessed impact of UK sugar tax on purchasing patterns [36].
Impact of events and economic shifts	
COVID-19 impact on fish and seafood purchases	Explored changes in fish, seafood, and related product purchases during COVID-19 lockdown [37].
Great Recession impact on food purchases	Analyzed effects of the Great Recession on UK food purchases [38].

#### Assessing Diet and Nutritional Inequalities

One major advantage of loyalty card data is its ability to reveal dietary disparities that traditional surveys often miss. For example, a study linking supermarket loyalty card data with a nutrient composition database found that lower-income households tended to purchase foods lower in fiber, a key marker of diet quality [20]. Similarly, spatial analyses have shown that fruit and vegetable purchases are more common in affluent areas and among older populations [21]. Such insights can inform targeted nutritional interventions.

Integrating food purchase records with health data and nutritional databases further expands the epidemiological applications of loyalty card data. To give one example, a study linking loyalty card data with medical prescription records found nutrient diversity and caloric intake to be the strongest predictors of “metabolic syndrome” diseases: hypertension, high cholesterol, and diabetes [34]. By enabling dynamic tracking of diet-disease relationships, these linkages overcome the limitations of survey-based dietary assessments, which provide only fragmented snapshots of exposure. Future research could explore how medical diagnoses, economic conditions, or life transitions shape food choices and associated health outcomes.

These data can also help identify population-level nutritional risks emerging from demographic and cultural shifts, providing early warnings for public health practitioners. For example, a loyalty card data study observed an association between transitioning to nondairy milk alternatives and iodine deficiency risk [24], while another revealed that older adults (aged 55+) in the United Kingdom were less likely to meet recommended levels of protein intake [25]. Importantly, loyalty card data have been shown to be a resource-efficient and moderately valid measure of dietary intake in large samples, reinforcing its value as a complementary tool to self-report surveys [26].

#### Policy and Intervention Evaluations

Loyalty card data serve as a powerful tool for evaluating public health policies and interventions. For example, they have been applied to assess the impact of fiscal measures—such as sugar-sweetened beverage taxes and the soft drinks sugar tax—on purchases of targeted items [35,36]. In addition, studies using loyalty card data have examined the effects of economic incentives such as fruit and vegetable vouchers [27], discounts [32], and points-plus-cash programs promoting healthier diets [28], as well as supermarket nudging strategies aimed at reducing ultraprocessed food consumption [29]. By tracking actual purchases of food items rather than relying on self-reported compliance, these data can facilitate more timely and objective evaluations of intervention or policy impact.

### Alcohol

Alcohol epidemiology faces challenges from falling survey response rates and inherent limits of traditional data sources [39]. Self-reported surveys are prone to recall bias and social desirability effects, leading to underreporting—particularly among heavy drinkers—while aggregate sales data lack the granularity to capture individual-level behaviors and demographic differences [40,41].

Loyalty card data provide a cost-effective supplement to traditional surveys by providing detailed, longitudinal records of alcohol purchases—including product type, quantity, and price. These passively collected data reduce respondent burden and self-reporting biases, allowing researchers to address key epidemiological questions: How do purchasing patterns vary by demographic and socioeconomic status? How can we identify seasonal, policy-driven, or life event–related shifts in alcohol consumption? How are these patterns associated with co-occurring risk behaviors and health outcomes?

A notable benefit of loyalty card data is the ability to continuously track alcohol purchase patterns over time, capturing shifts driven by seasonal variations [42], societal trends (eg, rising abstinence and declining alcohol use) [43] or policy changes [44]. For instance, a Finnish study used loyalty card data to evaluate the impact of a legislative reform allowing grocery stores to sell stronger alcoholic beverages, revealing distinct demographic differences in alcohol purchasing patterns [44]. Similar approaches could be used to assess the impact of policies (eg, minimum unit pricing) or abstinence campaigns (eg, Dry January or Febfast) to determine whether reductions in alcohol purchases persist or relapse over time. By continuously tracking purchases, these insights can enable epidemiologists to assess both immediate policy effects and longer-term behavioral shifts.

Loyalty card data also advance exposure modeling by capturing individual-level alcohol purchasing behaviors over time. A Finnish study demonstrated that beer purchase frequency aligned closely with self-reported beer drinking frequency, supporting the validity of purchase-based estimates of alcohol consumption [45]. Moreover, these data identify co-occurring risk behaviors often overlooked in analyses examining associations between alcohol consumption and health outcomes. This is illustrated by a Finnish study which showed that alcohol purchases frequently coincided with tobacco and unhealthy food purchases [46], while a French study revealed that wine buyers were more likely to purchase healthier foods, whereas beer consumers favored processed and high-fat items [47].

When linked with health records, loyalty card data can serve as a valuable tool to help identify purchasing patterns associated with at-risk populations. These insights can inform adaptive, evidence-based public health strategies, allowing interventions to be responsive to emerging trends in alcohol use and associated risks.

### Tobacco

Epidemiological surveys of tobacco consumption have traditionally focused on cigarette smoking, often overlooking the growing use of less regulated products, such as e-cigarettes, waterpipes, and smokeless tobacco [48]. This narrow scope creates blind spots in understanding the adoption, user demographics, and evolving consumption patterns of newer tobacco products, especially relevant amid the surge in e-cigarette use among young people [49]. Given the fast-changing landscape of tobacco products, more adaptive, real-time surveillance systems are urgently needed.

Loyalty card data, although underused in tobacco research [50], holds valuable information on category preferences, product characteristics (eg, nicotine strength), and purchasing frequency. These data can help address questions like: How are emerging tobacco products, including disposable e-cigarettes, being adopted across different age groups, socioeconomic strata, and geographical areas? How do purchasing behaviors respond to product innovations, industry marketing strategies, or local policy changes—and how are these shifts associated with downstream health outcomes or risk trajectories? To what extent do tobacco purchases co-occur with other risky behaviors—such as alcohol use or unhealthy food consumption—and how do these patterns vary across populations?

In addition, loyalty card data can reveal disparities in tobacco use—especially among marginalized communities targeted by industry marketing—and thereby inform regulatory decisions and public health strategies aimed at curbing emerging tobacco trends and associated risks [2].

### OTC Medications

Loyalty card data from supermarkets and pharmacies provide a continuous, high-frequency record of OTC medication purchases and can enable near real-time monitoring of self-medication behaviors, seasonal illness trends, and potential public health risks.

At the population level, loyalty card data can support passive syndromic surveillance by tracking OTC medication purchases. For example, spikes in purchases of pain relief, gastrointestinal, and respiratory treatments have been shown to predict seasonal illnesses, including influenza, up to 4 weeks in advance—outperforming traditional surveillance models [51]. Integrating OTC medication data into forecasting systems has also improved the accuracy of weekly forecasts of respiratory deaths in England, compared to models using only sociodemographic or weather variables [52]. In addition, these data reveal geographic and socioeconomic differences in OTC medication use. In England, cough and cold remedy purchases were higher in areas with greater exposure to air pollution (PM10 and NO2) [17], while analyses of pain relief, allergy treatments, and sun care products revealed notable geographic and income-based patterns [11]. When linked to health records, loyalty card data could further enhance early warning systems and strengthen disease forecasting models.

OTC medication purchase patterns may also serve as early indicators of disease, helping detect emerging preclinical health changes. Consider a case-control study which found that individuals diagnosed with ovarian cancer showed increased purchases of pain relief and indigestion remedies months before diagnosis, reflecting early, nonspecific symptoms [53]. Such findings suggest that loyalty card data could support early disease detection and inform targeted screening strategies.

Moreover, loyalty card data offer promising avenues for studying medication adherence and treatment continuity. For example, a loyalty card analysis found that frequent store visits and higher spending per trip were associated with better long-term statin adherence [54], while analyses of nicotine replacement therapy purchases revealed patterns of premature discontinuation**,** mirroring survey findings on cessation challenges [55]. By integrating prescription and loyalty card data, researchers may be able to refine interventions to improve adherence and treatment outcomes.

## Methodological Caveats

While loyalty card data hold promise for epidemiological research, a number of caveats must be addressed. Building on detailed discussions elsewhere [12,15], we group these into 3 themes: issues intrinsic to loyalty card data, considerations pertinent to epidemiologists, and challenges that arise when integrating with other data sources.

First, because loyalty card data are inherently personal—even when deidentified—robust data-sharing agreements are critical to avoid breaches and maintain public trust [9,34]. This raises broader ethical concerns around the secondary use of commercially collected data for public health research. Informed consent is rarely sought at the point of purchase, raising issues of transparency and autonomy. Data ownership remains ambiguous: while individuals generate the data, retailers control access and use, complicating accountability and benefit-sharing. Commercial partnerships may also create conflicts of interest if contractual terms influence data access, interpretation, or dissemination. Although a detailed discussion of ethical considerations around loyalty card data is beyond the scope of this paper, we encourage readers to consult papers addressing these concerns in detail [12,16,56,57].

From an epidemiological perspective, loyalty card datasets record purchasing—product category, price, quantity, timestamp, and so on—rather than consumption (see the “Variables captured in loyalty card data” column of Table 2 for specific fields available across behavioral domain). Because purchases do not always translate into intake, exposure misclassification is possible [18]. Table 2 summarizes the chief ways this misclassification can arise in each behavioral domain. In dietary assessments, discrepancies may emerge due to household sharing, food waste, and meals consumed outside the home [17]. Similarly, many alcohol purchases occur at off-licences or discount retailers that do not use loyalty programs, and tobacco sales may be underrepresented if products are bought at separate counters or are not linked to loyalty cards. In the case of OTC medications, regulatory changes—such as limits on stimulant laxatives or painkillers—can modify purchasing patterns independently of health needs, complicating trend analyses. Because of these gaps, loyalty card indicators require validation against established measures. Recent studies show this is feasible: LoCard [26] and STRIDE (Supermarket Transaction Records In Dietary Evaluation) [58] studies compared grocery purchases with food frequency questionnaires; a Switzerland-based study calibrated purchase-based diet scores against self-reported food intake [59]; the Supreme Nudge trial compared loyalty card data and self-reported diets in an intervention setting [60]; and a Finnish study found that beer purchase frequency estimated drinking frequency with fair to good accuracy, depending on how much participants used the same retailer [45]. Although purchases cannot reflect consumption perfectly, these validation studies demonstrate that well-chosen indicators can produce meaningful behavioral proxies. Future work should focus on identifying which product-level or category-level variables track well-established risk exposures most reliably, thereby reducing bias in population surveillance.

**Table 2.** Summary of research applications and methodological limitations of loyalty card data across 4 behavioral risk domains.

Behavioral domain	Variables captured in loyalty card data	Key methodological limitations
Diet	Types of food purchased (eg, fresh produce and processed foods), quantity, frequency of purchase, nutrient profile via linkage with food databases	Does not capture actual consumption; household-level purchases may not reflect individual intake; limited coverage of food wastage or food eaten at out-of-home sectors; often requires linkages to other databases to extract information on ingredients or nutritional content.
Alcohol	Product type (beer, wine, and spirits), quantity, timing, and price sensitivity	Under coverage of alcohol bought at off-licences or other retailers; purchases may not reflect consumption; limited product-level detail on alcohol content (ABV).
Tobacco	Product category (eg, cigarette, e-cigarette, or smokeless tobacco); product brand; product characteristics (eg, nicotine strength), price, purchase frequency, and quantity	Low volume of purchases captured due to separate counters or exclusion from loyalty schemes; may lack user demographics; regulatory changes may skew trends.
Over-the-counter medications	Product category (eg, pain relief medications, cough and cold remedies, allergy medicines, period products, laxatives, nicotine replacement therapy products, and supplements), quantity, frequency, and timing	Purchases may not indicate use; regulatory limits (eg, on stimulant laxatives or painkillers) can alter purchasing patterns independently of health needs.

Representativeness is another core limitation: not all consumers participate in loyalty programs, and some may pay with nontraceable methods or shop across multiple retailers, introducing selection bias [26]. Supermarket loyalty card data may overrepresent certain demographic groups, including older adults [20,21], females [60], smaller households [61], or those with higher education [21,22] and income [28,61] compared to the general population. This may lead to underrepresentation of vulnerable groups—failure to account for which could potentially skew behavioral estimates and mask or misrepresent the burden of risk among socioeconomically disadvantaged populations—compromising the validity of inferences about health inequalities. Future studies should consider weighting strategies [13], linkage to existing population datasets [16], or validation against population-representative samples to assess and correct for these biases [13].

Moreover, since loyalty card datasets are commercially produced, they may suffer from missing entries, inconsistent categorization, or insufficient detail in product groupings [17]. For epidemiologists, distinguishing between “risky” and “non-risky” consumption can become complicated when product categories are not clearly defined or standardized. Moreover, changes in retailer product categorizations and loyalty program structures can affect data consistency over time, complicating longitudinal analyses and necessitating regular model recalibration. Finally, causal inference may be particularly challenging: reverse causation can arise if individuals alter their purchasing patterns after a health diagnosis, while unmeasured confounders—such as local food environments or targeted marketing—can produce spurious associations [32]. To strengthen scope for causal interpretations, researchers must design studies with clearly defined hypotheses, reliable exposure indicators, and statistical methods that account for temporal variations and potential confounders [51].

From a technical standpoint, processing large-scale loyalty card data requires sophisticated preprocessing, substantial computational power, and statistical expertise to extract meaningful patterns while avoiding overfitting [62]. Advanced machine learning and statistical techniques are crucial for reconciling data inconsistencies, detecting outliers, and managing large-scale transactional datasets [62]; however, these methods often exceed the routine methodological toolkit of many epidemiologists, highlighting the need for interdisciplinary collaboration with data scientists and health informatics specialists [21]. By drawing on established protocols designed for loyalty card linkages with existing cohort studies [16], maintaining transparent governance structures, and fostering multisector collaborations, researchers can better leverage loyalty card data to generate insights into population health.

Importantly, realizing the potential of loyalty card data for epidemiological research requires integrating these data with existing cohort studies and health records. Such linkages demand careful handling to preserve privacy, ensure accurate matching, and mitigate risks of reidentification [14]. Inaccurate linkage can lead to spurious associations, while reliance on single-retailer datasets may provide only a partial view of health behaviors. Although linking data across retailers could provide richer insights, ensuring compatibility among disparate data systems and navigating strict data privacy laws remain significant hurdles [26].

Beyond loyalty card data integration with cohort studies and health records, future research should explore linking loyalty card data with emerging digital health sources. These include smartphone health apps (eg, diet tracking or menstrual cycle apps), wearable devices (eg, fitness trackers and sleep monitors), and even social media platforms (eg, posts or interactions reflecting mood or stress). Such linkages would enable more dynamic and multidimensional behavioral phenotyping—capturing not only what people buy but also when, why, and how health behaviors cluster in real time.

## Implications for Policy Makers and Public Health Agencies

Beyond academic research, loyalty card data can be embedded directly into routine public health intelligence systems. Because loyalty card data are generated daily, public health agencies may be able to construct near-real-time dashboards that flag unusual spikes in purchases of sentinel products—for example, analgesics or cough-and-cold remedies—as early warnings of respiratory outbreaks weeks before clinical reports become available [51,52]. At finer spatial scales, anonymized postcode-level purchase maps can reveal “hot spots” of risky behaviors (eg, clusters of high-strength alcohol purchases or disposable e-cigarette sales) that could enable local authorities to deploy targeted place-based health-promotion teams or licensing controls. Finally, because loyalty card data streams document population response to fiscal or regulatory actions (such as sugar-sweetened beverage taxes or alcohol price changes), policymakers can build rapid feedback loops that track equity-stratified effects within weeks rather than years and iteratively refine policies while they are still politically and economically tractable.

## Conclusions

Loyalty card data represent a transformative frontier for public health surveillance. By providing unprecedented granularity and scale in capturing real-world behaviors—diet, alcohol, tobacco, and OTC medication—these data can not only supplement but, in many cases, substantially enhance insights gained by traditional self-report methods. When linked with existing health records, loyalty card data have the potential to improve exposure measurement, enable dynamic risk modeling, and accelerate the evaluation of policies and public health interventions. Unlocking its full potential will demand bold investment in technical capacity, stronger data governance, and cross-sector collaboration. But the payoff is clear—a more agile and data-rich public health ecosystem capable of responding to emerging threats with greater speed and precision.

## Ethical Considerations

This viewpoint does not report new research involving human participants, animals, or identifiable personal data. All examples and datasets referenced are drawn from previously published sources already in the public domain; therefore, formal ethics committee review and informed consent were not required.

## Abbreviations

GDPR: General Data Protection Regulation
NCD: noncommunicable disease
OTC: over-the-counter
STRIDE: Supermarket Transaction Records In Dietary Evaluation
